# Efgartigimod for generalized myasthenia gravis: A multicenter real‐world cohort study in China

**DOI:** 10.1002/acn3.52142

**Published:** 2024-07-07

**Authors:** Sushan Luo, Qilong Jiang, Wenshuang Zeng, Qinzhou Wang, Zhangyu Zou, Yanyan Yu, Daojun Hong, Quantao Zeng, Song Tan, Zhouao Zhang, Yong Zhang, Xiuming Guo, Jing Chen, Zhongyan Zhao, Shixiong Huang, Jianquan Shi, Ying Chen, Lei Du, Chong Yan, Jianying Xi, Jie Song, Chongbo Zhao, the Chinese Myasthenia Gravis Collaborating Group (CMGCG), Yuzhou Guan, Yuwei Da, Huan Yang, Ting Chang, Yuge Wang, Huiyu Feng, Bitao Bu, Hongyu Zhou, Chunsheng Yang, Hua Zhang, Hui Deng, Ruisheng Duan

**Affiliations:** ^1^ Huashan Rare Disease Center and Department of Neurology, Huashan Hospital, Shanghai Medical College, National Center for Neurological Disorders Fudan University Shanghai 200040 China; ^2^ Department of Myopathy The First Affiliated Hospital of Guangzhou University of Chinese Medicine Guangzhou 510405 China; ^3^ Department of Neurology Hongkong University Shenzhen Hospital Shenzhen 518053 China; ^4^ Department of Neurology Qilu Hospital of Shandong University Jinan 250012 China; ^5^ Department of Neurology Fujian Medical University Union Hospital Fuzhou 350001 China; ^6^ Department of Neurology The First Affiliated Hospital of Nanchang University Nanchang 330006 China; ^7^ Department of Neurology, Sichuan Provincial People's Hospital University of Electronic Science and Technology of China Chengdu 610072 China; ^8^ Department of Neurology Affiliated Hospital of Xuzhou Medical University Xuzhou 221004 China; ^9^ Department of Neurology The First Affiliated Hospital of Chongqing Medical University Chongqing 400016 China; ^10^ Department of Neurology The Second Affiliated Hospital of Soochow University Suzhou 215004 China; ^11^ Department of Neurology, Hainan General Hospital Hainan Affiliated Hospital of Hainan Medical University Haikou 570311 China; ^12^ Department of Neurology, Nanjing First Hospital Nanjing Medical University Nanjing 210006 China; ^13^ Department of Neurology The First Affiliated Hospital of Wannan Medical College Wuhu 241004 China; ^14^ Department of Neurology The First Affiliated Hospital of Xinjiang Medical University Urumqi 830054 Xinjiang Uygur Autonomous Region China

## Abstract

**Objective:**

Efgartigimod, a neonatal Fc receptor antagonist, facilitates antibody degradation including pathogenic IgGs. The ADAPT study demonstrated the tolerability and efficacy of efgartigimod in the treatment of generalized myasthenia gravis (gMG). However, very limited evidence is available for the Chinese population, and it remains inconclusive about which kind of patients are selected to preferentially receive efgartigimod in real‐world settings.

**Methods:**

This multicenter cohort study included gMG patients treated at 14 neuromuscular reference centers in China. The Myasthenia Gravis Activities of Daily Living (MG‐ADL) score, immunosuppressants, and the incidence of treatment‐emergent adverse events (TEAEs) were prospectively collected.

**Results:**

Of the 1640 gMG admitted between September and December 2023, 61 (3.7%) received efgartigimod for at least one treatment cycle. Among them, 56 cases (92%) were anti‐AChR antibody‐positive, 4 were anti‐MuSK antibody‐positive, and 1 was seronegative. Thymoma‐associated myasthenia gravis accounted for most cases (44%, 27 out of 61). The principal causes of efgartigimod initiation included MG acute exacerbation (MGAE) (48%, 29 out of 61) and myasthenic crisis (MC) (15%, 9 out of 61). Clinically meaningful improvement was rapidly achieved in 97% (58 out of 61) of patients at 1.3 ± 0.7 weeks. By week 12, the MG‐ADL score reduced to 3.8 ± 4.1 (baseline:10.5 ± 5.2) for all participants, while it reduced to 4.0 ± 4.7 for MGAE and 3.8 ± 4.2 for MC, respectively. All but one TMG patient required no additional rescue therapies after efgartigimod initiation. 11.5% (7 out of 61) reported ≥1 TEAEs.

**Interpretation:**

This multicenter cohort study demonstrated the efficacy of efgartigimod in rapid control of gMG. Patients with MGAE or MC would benefit from efgartigimod treatment.

## Introduction

Myasthenia gravis (MG) represents an autoimmune disorder in which pathogenic immunoglobulin G (IgG) antibodies target the neuromuscular junction.^1^ Approximately 85% of MG cases are positive for anti‐acetylcholine receptor (AChR) autoantibodies. Other MG‐associated antibodies encompass anti‐muscle‐specific tyrosine kinase (MuSK) and anti‐lipoprotein receptor‐related protein 4 (LRP4) antibodies (2–5%).^1^ About 80% of all MG patients develop generalized MG (gMG),^2^, ^3^ which is further categorized as thymoma‐associated MG (TMG), AChR early‐onset MG (EOMG), AChR late‐onset MG (LOMG), MuSK‐MG, and seronegative MG (SNMG) according to recent advances in the understanding of disease mechanisms in MG.^4^ Even with the recommended therapies within the current treatment paradigm, gMG still imposes a heavy disease burden that is associated with unpredictable disease course and treatment process.^5^ Particularly, gMG cases with an active disease course have a greater risk of social disability and poor quality of life. Some patients present continuous symptoms with or without crisis or crisis‐like deterioration, as well as resistance to therapy.^6^ This highlights unmet needs in the therapeutic field of gMG.

Efgartigimod is a human IgG1's Fc fragment engineered for enhanced affinity to FcRn compared with endogenous IgG, thereby reducing IgG recycling and increasing IgG degradation. It is the first‐in‐class FcRn antagonist approved in the USA, Japan, and Europe, for the treatment of gMG based on the ADAPT study.^7^ Efgartigimod in China is used as an add‐on to standard therapy in adult gMG cases with anti‐AChR antibody positivity. Real‐world studies demonstrated clinically meaningful improvement (CMI) in Myasthenia Gravis‐Activities of Daily Living (MG‐ADL) not only in AChR‐gMG cases but also in multiple cases with MuSK‐ and triple‐negative gMG.^8^, ^9^, ^10^, ^11^ However, TMG and gMG cases with high disease activity only accounted for a small proportion in recent cohort studies. Further, the features of gMG cases that would choose efgartigimod remain unclear.

The present multicenter study aimed to analyze the indications of efgartigimod for gMG cases in real‐world practice in China. We hypothesized that efgartigimod would benefit gMG cases regardless of initiation status, acting for rapid disease control as well as maintenance therapy.

## Methods

### Patients and methods

Between September and December 2023, we followed 1640 gMG patients who were treated in 14 independent tertiary neuromuscular centers treating adult or adolescent MG from East, West, and South China. These hospitals were affiliated with Fudan University, Guangzhou University of Chinese Medicine, Hongkong University, Shandong University, Fujian Medical University, Nanchang University, University of Electronic Science and Technology of China, Xuzhou Medical University, Chongqing Medical University, Soochow University, Nanjing Medical University, Wannan Medical College, and Xinjiang Medical University. The diagnosis of MG was based on (1) onset symptoms and signs compatible with gMG; (2) detection of AChR or MuSK antibodies, or double‐seronegative but positive repetitive nerve stimuli (RNS).

Clinical status and disease severity were determined by the recommendations of the Myasthenia Gravis Foundation of America (MGFA) and German guidelines. TMG was defined as gMG with a history of thymoma including all ages. EOMG was AChR‐positive generalized MG without thymoma, with onset age <50 years. LOMG was AChR‐positive generalized MG without thymoma, with onset age ≥50 years. MuSK‐MG was a generalized MG with positive MuSK antibodies. SNMG cases were individuals double‐negative for AChR and MuSK antibodies. MG acute exacerbation (MGAE) was defined as worsening of MG symptoms, including dysphagia, dyspnea, and decline in general limb muscle strength requiring monitoring or treatment.^12^ Myasthenic crisis (MC) was defined as a rapid clinical decline in respiratory function requiring noninvasive ventilation or intubation with mechanical ventilation (MV). Maintenance therapies with intravenous immunoglobulin (IVIg) or therapeutic plasma exchange (TPE) were defined as repeated IVIg and/or TPE for the last 3 months.

All clinical data were collected after signed informed consent. The study protocol was specifically approved by the institutional review board of the ethics committee of each institution (Fudan University, Huashan Hospital, IRB 2023‐1100). The clinical study followed the Declaration of Helsinki.

### Efgartigimod administration and prospective follow‐up

Efgartigimod was administered intravenously at 10 mg/kg as a 1‐h infusion in cycles of four weekly infusions. In the subsequent flexible cyclical period, efgartigimod was readministered as four weekly infusions at the discretion of the treating physician in case of acute worsening of MG‐ADL.

Baseline parameters were retrospectively analyzed, including sex, age, disease course, serum antibody type, gMG subtypes (TMG, EOMG, LOMG, MuSK‐MG, and SNMG), history of thymectomy, treatments for MG before efgartigimod administration, prednisone dose, Myasthenia Gravis Activities of Daily Living (MG‐ADL) score, MGFA classification, and the indications of efgartigimod as an add‐on therapy.

After the initiation of efgartigimod administration, therapeutic response based on MG‐ADL score and safety profile were prospectively recorded weekly for 12 weeks. Changes in prednisone dose and other immunotherapies were prospectively recorded at 4, 8, and 12 weeks after efgartigimod treatment initiation. Clinical meaningful improvement (CMI) was defined as ≥2‐point reduction of MG‐ADL score from baseline values. Minimal symptom expression (MSE) was defined as an MG‐ADL score of 0 or 1.^13^ Since baseline MG‐ADL scores differ in MG subtypes, “change in MG‐ADL” (average MG‐ADL reduction/baseline ADL) was used to evaluate therapeutic response, instead of the absolute value. For those who initiated the second cycle of infused efgartigimod, the therapeutic response and safety profile were still collected weekly till 12 weeks.

### Statistical analysis

Continuous variables were reported as mean (SD) and compared by independent t‐test or one‐way analysis of variance (ANOVA). Categorical data were reported as percentage (%) and compared by the chi‐squared test. The rstatix package in R version 4.03 (R Foundation for Statistical Computing) was used for data analysis. Figures were generated with tidyverse, ggpubr, and ggprism, also in R version 4.03.

## Results

### Baseline clinical features

Of 1640 gMG patients treated at 14 hospitals, 3.7% (61 patients) received at least one cycle of efgartigimod (male to female: 18 to 43). Baseline clinical characteristics are shown in Table 1. The mean patient age at admission was 55.3 ± 16.4 years (range: 17–86) and the mean disease duration from symptom onset to admission was 5.1 ± 8.2 years (range: 0–48). This multicenter cohort included 14 EOMG (23.0%), 15 LOMG (24.6%), 27 TMG (44.3%), 4 MuSK‐MG (6.6%), and 1 SNMG (1.6%). The gMG patients administered thymectomy (45.9%, 28 out of 61) included 6 thymic hyperplasia and 22 thymoma cases. IgG level at baseline was 13.8 ± 8.0 g/L (*n* = 27).

**Table 1:** Baseline clinical features for Chinese gMG patients who were treated with efgartigimod, total *n* = 61.

Clinical variables	Mean ± SD (range) or No.(%)
Age (years old)	55.3 ± 16.4
Sex (female%)	43 (70.5%)
Thymoma (%)	27 (44.3%)
Thymectomy (%)	28 (45.9%)
Onset age (years)	50.5 ± 18.1
Disease duration (years)	5.1 ± 8.2
MGFA classification at onset	
I	8 (13.1%)
II	17 (27.9%)
III	21 (34.4%)
IV	12 (19.7%)
V	3 (4.9%)
Clinical classification with efgartigimod initiation	
EOMG	14 (23.0%)
LOMG	15 (24.6%)
TMG	27 (44.3%)
MUSK‐MG	4 (6.6%)
SNMG	1 (1.6%)
Previous treatment	
Pyridostigmine	56 (91.8%)
Prednisone	54 (88.5%)
Tacrolimus	26 (42.6%)
Azathioprine	2 (3.3%)
Mycophenolate mofetil	9 (14.8%)
Cyclophosphamide	0 (0%)
Rituximab	5 (8.2%)
Long‐term sustained IVIg/PE	8 (13.1%)
MGFA classification with efgartigimod initiation	
I	0
II	14 (23.0%)
III	22 (36.1%)
IV	16 (26.2%)
V	9 (14.8%)
Initiation status	
MGAE	29 (47.5%)
MC	9 (14.8%)
Mild/moderate	23 (37.7%)
Prednisone dosage at baseline (mg/d)	28.3 ± 23.0

Baseline refers to the first infusion of efgartigimod. Disease duration was defined as the time between onset symptom and the baseline entry.

EOMG, early‐onset MG; IVIg, intravenous immunoglobulin; LOMG, late‐onset MG; MC, myasthenic crisis.; MGAE, MG acute exacerbation; MGFA, Myasthenia Gravis Foundation of America; MuSK‐MG, muscle‐specific tyrosine kinase; PE, plasma exchange; SNMG, seronegative MG; TMG, thymoma‐associated MG.

Before efgartigimod initiation, pyridostigmine was employed in 91.8% (56 out of 61) of cases. Immunosuppressants (IS) included glucocorticoids (54 out of 61, 88.5%), tacrolimus (26 out of 61, 42.6%), azathioprine (2 out of 61, 3.3%), mycophenolate mofetil (9 out of 61, 14.8%), rituximab (5 out of 61, 8.2%), and maintenance therapies including IVIg and TPE (8 out of 61, 13.1%). MGFA classes at onset included MGFA I (13.1%, 8 out of 61), MGFA II (27.9%, 17 out of 61), MGFA III (34.4%, 21/61), MGFA IV (19.7%, 12 out of 61), and MGFA V (4.9%, 3 out of 61).

The indications of efgartigimod initiation included a highly active disease (62.3% [38 out of 61], including MGAE [47.5%, 29 out of 61] and MC [14.8%, 9 out of 61]) and a mild/moderate disease (37.7%, 23 out of 61). If assessed by MGFA class, there were 14 MGFA II (23.0%), 22 MGFA III (36.1%), 16 MGFA IV (26.2%), and 9 MGFA V (14.8%) cases.

### Overall efficacy and safety

The efficacy indexes in gMG are listed in Table 2. In the whole cohort, baseline mean MG‐ADL score was 10.5 ± 5.2, and average prednisolone dose was 28.3 ± 23.0 mg. Under efgartigimod treatment, CMI was rapidly achieved in 97% (58 out of 61) of patients, with a mean time of 1.3 ± 0.7 weeks. By week 12, 39% (23 out of 59) of cases achieved MSE while MG‐ADL was reduced to 3.8 ± 4.1. The change in MG‐ADL reached 59.9 ± 53.7% by week 12, compared to baseline values. In patients on prednisone through efgartigimod treatment (*n* = 50), the average daily dose of prednisolone was reduced to 22.3 ± 19.0 mg by week 4, 19.1 ± 16.7 mg by week 8, and 15.7 ± 14.8 mg by week 12. After efgartigimod initiation, add‐on immunotherapies were administered, including tacrolimus (*n* = 6), rituximab (*n* = 2), and mycophenolate mofetil (*n* = 2). The second cycle of efgartigimod was initiated in 20% (12 out of 61) of patients, with a mean interval of 42 days, including 4 EOMG, 1 LOMG, and 7 TMG cases.

**Table 2:** The efficacy profile of efgartigimod in gMG patients, total *n* = 61.

Group	Time to CMI (w)	Baseline MG‐ADL (MSE%)	4w MG‐ADL (MSE%)	8w MG‐ADL (MSE%)	12w MG‐ADL (MSE%)	Change in MG‐ADL (W4)	Change in MG‐ADL (W8)	Change in MG‐ADL (W12)
All patients (*n* = 61)	1.3 ± 0.7	10.5 ± 5.2 (0%)	3.2 ± 4.5 (45.9%)	3.9 ± 4.3 (32.8%)	3.8 ± 4.1 (39.0%)	73.1% ± 36.9%	61.1% ± 53.3%	59.9% ± 53.7%
AChR‐Ab+								
EOMG (*n* = 14)	1.3 ± 0.5	10.9 ± 6.1 (0%)	3.3 ± 4.9 (57.1%)	4.6 ± 3.8 (21.4%)	4.5 ± 3.6 (14.3%)	79.8% ± 26.2%	56.8% ± 30.5%	48.8% ± 36.3%
LOMG (*n* = 15)	1.1 ± 0.4	8.8 ± 3.8 (0%)	1.9 ± 2.2 (46.7%)	2.3 ± 3.2 (53.3%)	2.1 ± 2.9 (60.0%)	79.0% ± 20.3%	76.8% ± 24.9%	80.2% ± 20.2%
TMG (*n* = 27)	1.4 ± 1.0	10.7 ± 5.4 (0%)	4.1 ± 5.5 (37.0%)	4.7 ± 5.2 (25.9%)	4.5 ± 4.9 (37.0%)	63.6% ± 48.5%	49.8% ± 72.8%	50.0% ± 71.2%
AChR‐Ab−								
MUSK (*n* = 4)	1.0 ± 0	13.0 ± 6.1 (0%)	1.0 ± 1.4 (33.3%)	1.5 ± 1.9 (50%)	1.5 ± 1.9 (50%)	90.1% ± 15.9%	91.9% ± 9.9%	91.9% ± 9.9%
SNMG (*n* = 1)	1.0 ± 0	15.0 ± 0 (0%)	3.0 ± 0 (0%)	5.0 ± 0 (0%)	6.0 ± 0 (0%)	80% ± 0%	66.7% ± 0%	60.0% ± 0%

CMI refers to a reduction of at least a two‐point from the baseline MG‐ADL score. Minimal symptom expression (MSE) was defined as an MG‐ADL score of 0 or 1. “4w MG‐ADL,” “8w MG‐ADL,” and “12w MG‐ADL” were defined as the average ADL score from the initiation of efgartigimod to the 4th week, 8th week, and 12th week after treatment, respectively. “Change in MG‐ADL” was defined as the average ADL reduction/baseline ADL.

CMI, clinically meaningful improvement; MG‐ADL, Myasthenia Gravis‐Activities of Daily Living; MSE, minimal symptom expression.

The safety profile was generally good. A total of 9.8% (6 out of 61) of cases reported mild‐to‐moderate TEAEs, with the commonest being infections (2 patients had upper respiratory tract infection and 1 had herpes zoster infection in the neck), which were resolved by treatment with antimicrobials. Three patients reported transient muscle stiffness for 1 or 2 days after each infusion. Of note, 1 TMG patient had MGAE and subsequently developed MC requiring rescue therapies and intensive care.

### Subgroup analysis of therapeutic response

#### Efgartigimod in patients with distinct clinical subtypes

The time to achieve CMI was 1.3 ± 0.5 weeks and 1.4 ± 1.0 weeks for EOMG and TMG cases, respectively. By week 4, MSE was achieved in 57.1% and 37.0% of EOMG and TMG cases, respectively; however, by week 12, the proportion with MSE was reduced to 14.3% and 37.0%, respectively. Patients with LOMG had the mildest baseline severity, with an average MG‐ADL score of 8.8 ± 3.8. MG‐ADL scores decreased to 2.1 ± 2.9, with 60.0% achieving MSE, by week 12.

**Figure 1:**
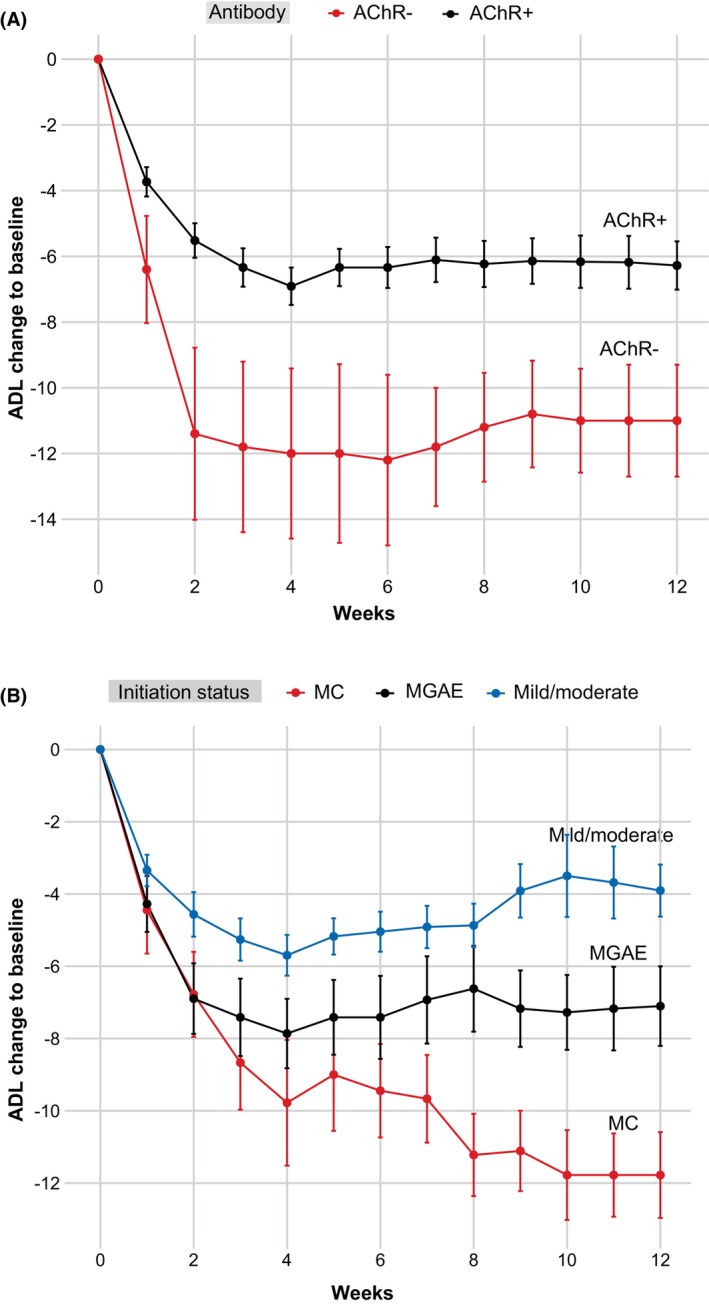
The changes in MG‐ADL scores of gMG stratified by the serotypes of antibody and initiation disease status. MG‐ADL score changes in AChR+ gMG (*n* = 56) and AChR− gMG (*n* = 5) (A). MG‐ADL score changes in gMG with mild/moderate disease (*n* = 23), MGAE (*n* = 29), and MC (*n* = 9) (B). AChR, acetylcholine receptor; gMG, generalized myasthenia gravis; MC, myasthenic crisis; MG‐ADL, Myasthenia Gravis Activities of Daily Living; MGAE, myasthenia gravis acute exacerbation.

Although MuSK‐MG and SNMG cases had relatively high baseline MG‐ADL scores (13.0 and 15.0, respectively), they achieved CMI within 1 week. By week 4, MuSK‐MG cases had an average MG‐ADL score of 1.0 ± 1.4, with 33.3% achieving MSE, while SNMG cases had an average MG‐ADL score of 3.0. By week 12, the average MG‐ADL score was reduced to 1.5 ± 1.9 for MuSK‐MG, with 50% of patients having MSE, while it increased to 6.0 for the SNMG case (Fig. 1A).

The change in MG‐ADL score was used to assess the subjective therapeutic response for each clinical subtype. MuSK‐MG cases had 90.1 ± 15.9% and 91.9% ± 9.9% reductions by weeks 4 and 12, respectively, representing the best responder among all subtypes. No MuSK‐MG case requested or required a second cycle of efgartigimod treatment. In contrast, TMG cases exhibited 63.6 ± 48.5% (week 4) and 49.8 ± 72.8% (week 12) MG‐ADL score reductions compared with baseline, indicating a less favorable response.

#### Efgartigimod efficacy in patients at different initiation status

We next stratified the participants by the initiation status. By week 4, MSE was achieved in 12 out of 23 (52.2%) patients with mild/moderate status, 13 out of 29 (44.8%) with MGAE, and 3 out of 9 (33.3%) with MC. By week 12, MSE was achieved in 9 out of 23 (39.1%) patients with mild/moderate status, 10 out of 29 (34.5%) with MGAE, and 4 out of 9 (44.4%) with MC (Fig. 1B). MG‐ADL scores in patients with MGAE were 3.3 ± 4.3 and 4.0 ± 4.7 by weeks 4 and 12, respectively (baseline: 11.1 ± 4.9). MG‐ADL scores in patients with MC reduced to 5.8 ± 7.4 and 3.8 ± 4.2 by weeks 4 and 12, respectively, (baseline: 15.6 ± 5.2).

**Figure 2:**
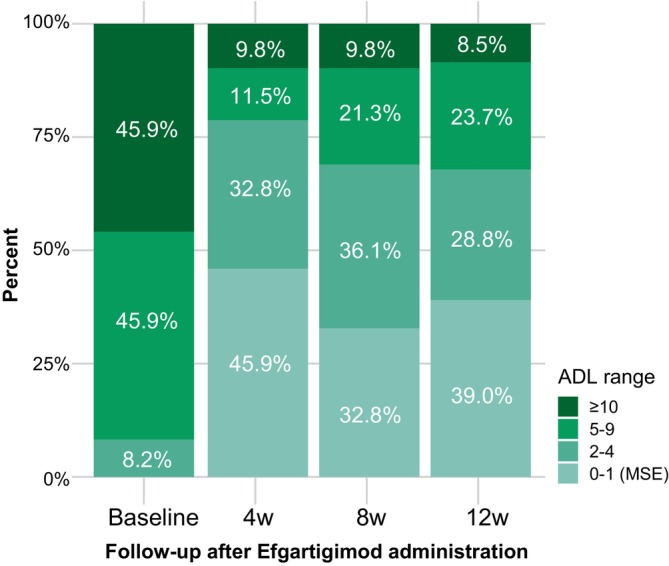
The proportion of gMG patients with different MG‐ADL scores at baseline, 4 weeks, 8 weeks, and 12 weeks after efgartigimod initiation. gMG, generalized myasthenia gravis; MG‐ADL, Myasthenia Gravis Activities of Daily Living.

We also stratified the participants by clinical severity according to baseline MG‐ADL score (Fig. 2). The proportion of gMG patients with high clinical severity (MG‐ADL≥10) decreased from 45.9% at baseline to 9.8% and 8.5% by weeks 4 and 12, respectively. The proportion of gMG cases with MSE increased from 0% at baseline to 45.9% by week 4, versus 39.0% by week 12. Approximately 70% of all participants with baseline MG‐ADL≥10 had a mild disease status, with MG‐ADL≤4.

#### Clinical profiles of patients with inadequate rapid response to efgartigimod

In this multicenter prospective cohort, 3 patients still did not achieve CMI by week 4 (4.9%). The clinical profiles and therapeutic responses of these patients are retrospectively reviewed below.

**Figure 3:**
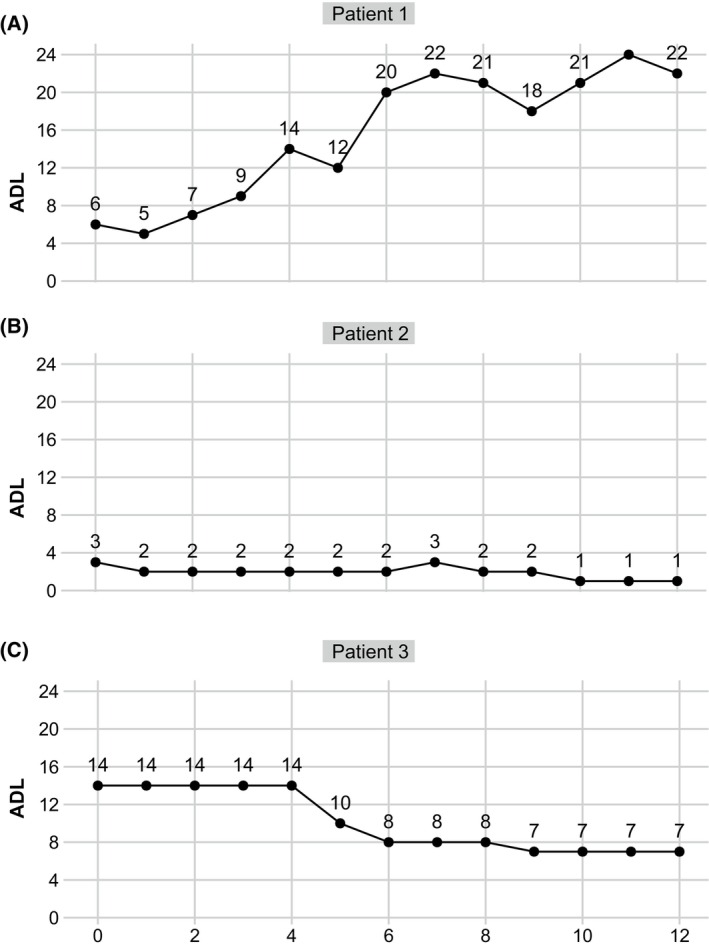
MG‐ADL score changes in 3 patients who did not exhibit rapid response to efgartigimod within 4 weeks. MG‐ADL, Myasthenia Gravis Activities of Daily Living.

*Patient 1* (TMG with acute worsening triggered by COVID‐19 infection): a 46‐year‐old female with AChR antibody‐positive gMG for 14 years. A thymectomy carried out in 2017 confirmed the pathology of thymoma B1. She experienced recurrent MC and was refractory to conventional immunotherapies. Rituximab was administered, resulting in stable disease with MSE since January 2023. Despite full vaccination, she had COVID‐19 infection in August 2023 and developed acute worsening 1 month later. Pulmonary CT revealed no pneumonia, thymoma metastasis or relapse. At admission, the patient was administered efgartigimod infusions for one cycle from September 2023. However, she gradually developed MC, requiring intensive care and plasma exchange (five cycles), followed by repeated IVIg. By the latest visit at week 12, this patient was still on ventilation (Fig. 3A).

*Patient 2* (Mild LOMG): A 73‐year‐old female with mild gMG for 4 months. She reported a recent exacerbation of ptosis of the left eyelid that impaired her quality of life (ADL = 3, with 2 points in ptosis, and 1 point in the upper limb). She declined a dose increase for daily oral prednisolone (40 mg) as well as other immunosuppressants as add‐on therapies. Thus, one cycle of efgartigimod was administered. However, 1 point change was achieved in upper limbs with no improvement in ptosis (Fig. 3B).

*Patient 3* (TMG with unresectable thymoma): A 64‐year‐old female, anti‐AChR antibody‐positive, complained of acute worsening in limbs for 3 months. A thymectomy carried out in 2020 confirmed the pathology of thymoma B3. She had recurrent proximal limb weakness for the past 2 years, and lung metastasis was detected by lung CT during regular visits. Before she could be safely transferred for chemotherapy, the patient was administered one cycle of efgartigimod and an escalation in the daily dose of prednisolone. Although the patient did not achieve CMI by week 4, the MG‐ADL score showed a progressive decrease from week 6 to a minimum score of 7.0 by week 12 (Fig. 3C).

## Discussion

The current study analyzed the indications of efgartigimod in adult Chinese patients in real‐world settings soon after its approval in China. The patients encompassed a majority of gMG cases with high disease activity including individuals with MGAE and MC, which were not adequately included in the ADAPT and ADAPT+ studies. This analysis also allowed a preliminary assessment of efgartigimod efficacy in gMG patients stratified by subtypes, and disease severity at initiation.

The emergence of new biologics provides opportunities in the field of MG to move from conventional treatments to target‐specific immunotherapies with less side effects and faster action. Eculizumab, a C5 inhibitor, as the first approved biologic for the treatment of AChR‐subtype gMG, was indicated in only 0.9% of all patients with gMG within 1 year since its approval in Japan.^14^ As the second approved biologic for gMG treatment, efgartigimod was used in about 3.7% of all gMG treated in the 14 participating Chinese centers. The indicated patients encompassed not only cases with highly active gMG despite adequate disease‐modifying and symptomatic therapies, but also those with a mild/moderate disease course with side effects or contradictions to conventional therapies. This represents a wide spectrum for efgartigimod in the treatment of gMG patients in real‐world settings.

In comparison with participants in a US multicenter cohort, gMG cases in this study had a more severe clinical status as reflected by higher baseline MG‐ADL scores (10.5 ± 5.2).^10^ However, the therapeutic response to efgartigimod was better in the Chinese cohort if assessed by the proportion of cases achieving CMI (97% vs. 72%) or MSE (45.9% vs. 25%) after one cycle of efgartigimod treatment. The potential explanation for this difference is a relatively younger admission age (55.3 ± 16.4 vs. 65.56 ± 14.74 years) and a higher proportion of thymectomy cases (45.9% vs. 18.9%) in this cohort. Further, a short disease duration (5.1 ± 8.2 years) from the disease onset to the efgartigimod initiation may have contributions in the better therapeutic response to efgartigimod from our cohort, as compared with the previous cohort studies and phase III ADAPT trial. Future cohort studies focusing on one particular gMG subtype are expected to explore the therapeutic response.

This analysis revealed beneficial effects for efgartigimod in gMG regardless of antibody type. Noticeably, MuSK‐MG also achieved a satisfactory MG‐ADL reduction after treatment, corroborating the ADAPT+ study and an Italian multicenter cohort study.^9^, ^15^ AChR‐MG follows a Th1 paradigm and is characterized by immunoglobulin G1 (IgG1) and IgG3 subclasses, which have effector functions that promote tissue damage at neuromuscular junctions (NMJ). There is a disconnect between disease severity and autoantibody titers. Conversely, MuSK MG relies on a Th2 response, which is largely governed by the IgG4 subclass that mediates pathological processes by physically blocking NMJ protein–protein interactions.^16^ Despite the abovementioned immunopathological discrepancy between the AChR and MuSK subtypes, efgartigimod showed equal efficacy in clearing pathogenic IgGs in IgG1, IgG3, and IgG4.

Even in cases positive for anti‐AChR antibodies, EOMG and TMG exhibited less favorable responses to efgartigimod as evidenced by a more pronounced MG‐ADL score rebound by week 8. EOMG and TMG cases accounted for the majority of patients administered the second cycle of efgartigimod, with an interval of 40 days from the first cycle, in which thymic inflammation has a profound effect on immunopathogenesis.^1^, ^17^ A hallmark of EOMG is thymic follicular hyperplasia, which encompasses germinal centers and an increased number of B cells and plasma cells and correlates with intrathymic production of heterogeneous IgG autoantibodies targeting AChR. The MGTX trial reported a beneficial effect for thymectomy in adult nonthymomatous MG cases.^18^, ^19^ As for the role of thymoma in the development of TMG, overgeneration and escape of Th cells from thymoma further induce B cells to produce autoantibodies against AChR in the periphery. Once this is initiated, skeletal muscle‐derived AChR/autoantibody complexes present in regional lymph nodes perpetuate TMG even after thymoma removal.^20^, ^21^ In this regard, further studies enrolling EOMG or TMG patients with larger samples and applying more infusion cycles are warranted to evaluate the efficacy of targeting biologics in these specific subtypes.

This retrospective analysis reported a TMG case who developed MC after COVID‐19 infection despite timely initiation of efgartigimod treatment. This points to an exacerbated immune response to acute respiratory syndrome coronavirus 2 (SARS‐CoV‐2). Indeed, COVID‐19‐mediated autoimmunity is associated with cytopathic effects by macrophages, CD8^+^ T cells and inflammatory monocytes,^22^ which may not be easily suppressed by conventional immunotherapies or a single cycle of rescue therapy.

This study had strengths. It included a relatively large sample size for a real‐world study of Chinese gMG patients. This cohort study allowed a preliminary assessment of efgartigimod's indications as well as therapeutic responses stratified by MG subtype and initiation status based on disease activity. Data for MGAE and MC cases further confirmed the roles of FcRn antagonists in fast‐acting therapies for gMG.

The limitations of this study included a short follow‐up period. In addition, patients administered the second cycle of efgartigimod were limited during the observation period. The repeated measurement of serum biomarkers, for example, pathogenic antibody or IgG levels, was unavailable in this real‐world study.

## Conclusion

This study provided the efficacy, safety, and tolerability data of efgartigimod in a Chinese multicenter real‐world cohort with an elevated proportion of cases with high disease activity. As MG treatment is shifting toward target‐specific biologicals, further prospective studies are warranted to examine if a given agent should be maintained or switched to another, as well as long‐term safety profiles.

## Funding Information

This work was supported by financial grants from the National Key Research and Development Plan of China (2022YFC3501303 and 2022YFC3501305) and the National Natural Science Foundation of China (82071410 and 82001335).

## Conflict of Interest

The authors declare that the research was conducted in the absence of any commercial or financial relationships that could be construed as a potential conflict of interest.

## Author Contributions

Conceptualization: Luo S and Zhao C; Investigation: Wang Q, Zou Z, Yu Y, Hong D, Zeng Q, Tan S, Zhang Z, Zhang Y, Guo X, Chen J, Zhao Z, Huang S, Chen Y, Du L, Yan C, Song J, and Xi J; Writing—original draft preparation: Luo S, Jiang Q, and Zeng W; Writing—review and editing: Shi J and Zhao C. All authors read and approved the final manuscript.

## Data Availability

Anonymized data will be made available upon reasonable request.
