# Correction to “Efgartigimod for Generalized Myasthenia Gravis: A Multicenter Real‐World Cohort Study in China”

**DOI:** 10.1002/acn3.70126

**Published:** 2025-07-01

S. Luo, Q. Jiang, W. Zeng, et al., “Efgartigimod for Generalized Myasthenia Gravis: A Multicenter Real‐world Cohort Study in China,” *Annals of Clinical and Translational Neurology* 11, no. 8 (2024): 2212–2221, https://doi.org/10.1002/acn3.52142.

1. Abstract—Results: Line 8, Clinically meaningful improvement was rapidly achieved in 97% (58 out of 61). This should be read as 95% (58 out of 61).
2. Results—Overall efficacy and safety: Line 5, 97% (58 out of 61). This should be corrected as 95% (58 out of 61).

We apologize for this error.

